# Supplementary material for: Depth-dependent dive kinematics suggest cost-efficient foraging strategies by tiger sharks
Source: R Soc Open Sci. 2020 Aug 19;7(8):200789. doi: 10.1098/rsos.200789 (PMC7481696; doi:10.1098/rsos.200789)
Supplement: Supplementary methods and results [file rsos200789supp1.pdf]

Supplementary Information

Depth dependent dive kinematics suggest cost-efficient foraging strategies by tiger sharks

Royal Society Open Science

Samantha Andrzejaczek[1,2*§], Adrian C. Gleiss[3], Karissa Lear[3], Charitha Pattiaratchi[1], Taylor Chapple[4] and Mark Meekan[2]

[1]*Ocean Graduate School & The UWA Oceans Institute, The University of Western Australia, Crawley, WA, 6009, Australia*
[2]*The Australian Institute of Marine Science, Crawley, WA, 6009, Australia*
[3]*Centre for Sustainable Aquatic Ecosystems, Harry Butler Institute, Murdoch University, Murdoch, WA, 6150, Australia*
[4]*Hopkins Marine Station, Stanford University, Pacific Grove, CA, 93950, US*
[*]*Present address: Hopkins Marine Station, Stanford University, Pacific Grove, CA, 93950, USA*
[§]*Author for correspondence: sandrzejaczek@gmail.com*

Table S1. Summary details of tagged tiger sharks. CC and CD in Tag ID refer to CATS Camera and CATS Diary tags respectively. *Tag malfunction, no data downloaded **Tag shut off before detachment [n]Resight – same as TS17.

Tiger shark ID	Tag ID	Deployment date	Galvanic timed release deployed	Attachment duration	Pre-caudal length (cm)	Fork length (cm)	Total length (cm)	Girth (cm)	Sex	Recovery latitude (°S)	Recovery longitude (°E)	Mean (± SD) depth (m)	Maximum depth (m)
**TS1**	CC1	23/4/2017 11:33	A4 – 15 hours	9h 51min	254	286	347	169	F	22.91	113.81	6.61 ± 4.39	17.49
**TS2**	CC2	23/4/2017 13:47	A4 – 15 hours	13h 14min**	272	311	345	NA	F	22.93	113.57	9.37 ± 5.89	34.45
**TS3**	CD1	23/4/2017 14:49	A4 – 15 hours	4h 41min	250	224	266	122	F	23.06	113.79	9.08 ± 3.59	18.61
**TS4**	CC1	26/4/2017 10:14	A4 – 15 hours	11h 20min	264.5	289	331	169	F	22.99	113.79	9.01 ± 3.41	17.21
**TS5**	CD2	26/4/2017 11:07	A4 – 15 hours	11h 37min	NA	NA	~350	NA	F	23.06	113.74	6.77 ± 4.23	19.17
**TS6**	CD1	26/4/2017 12:50	A4 – 15 hours	10h 24 min	240	253	300	150	F	23.04	113.78	8.01 ± 3.74	16.07
**TS8**	CC2	28/4/2017 12:01	A6 – 25 hours	17h 14min	260	283	321	159	F	23.06	113.78	8.78 ± 4.54	20.91
**TS9**	CC1	28/4/2017 12:40	A4 – 15 hours	9h 10min	293	314	345	NA	F	22.98	113.62	21.10 ± 24.99	74.33
**TS10**	CD2	28/4/2017 14:12	A4 – 15 hours	9h 52min	283	312	362	142	UN	23.05	113.80	8.09 ± 4.35	17.90

**TS11***	CD2	30/4/2017 13:05	A6 – 25 hours	NA	257	284	336	127	M	23.05	113.60	NA	
**TS12**	CC2	30/4/2017 13:50	A6 – 25 hours	13h 48min	301	332	380	181	F	23.07	113.74	5.95 ± 4.62	27.51
**TS13**	CD1	30/4/2017 14:37	A6 – 25 hours	20h 15min	215	229	277	119	F	22.91	113.76	28.08 ± 19.78	83.86
**TS14**	CC1	30/4/2017 15:13	A6 – 25 hours	17h 32min	267	299	351	167	M	22.96	113.81	4.09 ± 3.92	17.75
**TS15**	CC1	2/5/2017 12:19	C5 – 40 hours	48h 44min	270	298	329	161	F	22.76	113.70	7.08 ± 5.41	32.83
**TS16**	CD1	3/5/2017 9:19	A6 – 25 hours	17h 29min	202	223	268	108	F	22.87	113.77	4.07 ± 3.09	18.04
**TS17**	CC2	3/5/2017 9:35	A6 – 25 hours	15h 37min	297	323	373	171	F	22.93	113.77	4.54 ± 3.47	17.64
**TS18**	CC2	7/5/2017 10:31	A6 – 25 hours	16h 6min	270	300	330	NA	F	23.04	113.51	43.67 ± 31.44	93.91
**TS19**	CD1	7/5/2017 13:40	A6 – 25 hours	15h 10min	224	252	299	140	F	23.04	113.81	3.36 ± 3.89	17.28
**TS20**	CC1	7/5/2017 13:58	A6 – 25 hours	10h 38min	276	303	346	NA	F	22.93	113.80	2.703 ± 2.77	15.75
**TS24**[n]	CC2	14/5/2017 12:08	C5 – 40 hours	23h 43min	300	330	373	171	F	22.73	113.73	2.77 ± 3.46	17.78
**TS25**	CC2	18/5/2017 11:31	B5 – 32 hours	5h 7min**	201	223	265	104	F	22.86	113.65	7.06 ± 3.19	14.95
**TS27**	CC1	18/5/2017 14:31	A6 – 25 hours	13h 54min	NA	322	370	133	F	22.91	113.76	23.62 ± 21.39	72.79

Supplementary methods:

Data processing:

Depth record:

Vertical velocity (VV), defined as the rate of change in depth over a one second period, was used to split the depth record into vertical swimming phases (“ascending”, “descending” and “level swimming”). This was executed by smoothing the depth record using a 10 s running mean and calculating the average VV by taking the difference of this smoothed depth between successive points at 1 s intervals. Ascents and descents were defined where VV exceeded an absolute value of 0.05 m/s for more than 10 s, and level where this value was not exceeded [1, 2]. As error in the depth sensor was minimal (±10 cm), we do not believe sensor accuracy significantly affected vertical movement phase classification.

Tri-axial sensor data:

Data recorded by the accelerometer (acceleration) and gyroscope (angular velocity) were processed using Igor Pro ver. 7.0.4.1 (Wavemetrics, Inc. Lake Oswego, USA) and Ethographer [3]. The gravitational component of acceleration (static acceleration) was determined using a three-second box smoothing window on the raw acceleration data [4]. Shark body pitch angles (orientation of the shark with regard to the horizontal plane) were derived by calculating the arcsine of the static acceleration in the surging (posterior– anterior) axis. To correct for the tag attachment angle on each individual shark, we determined the pitch when the shark was swimming at a constant depth (when vertical velocity (VV) was equal to zero), and subtracted this value from all pitch estimates [5]. The dynamic component of acceleration was calculated by subtracting the gravitational component from the raw acceleration for each axis. We then used a continuous wavelet transformation on the dynamic component of the sway (lateral) axis to calculate the acceleration signal amplitude and frequency of tailbeats. Using these same methods, amplitude and frequency were calculated using the angular velocity data, and the resulting signals were compared with those derived from the acceleration data to determine the best measure of tailbeat kinematics. The angular velocity data produced the clearest tailbeat signal and consequently was used to quantify tailbeat kinematics and the incidence of gliding behavior (cessation of tailbeats for more than 1 s; see section below).

ODBA (overall dynamic body acceleration) was calculated by summing the absolute value of dynamic acceleration from all three axes [6].

Depth record

Vertical velocity (VV), defined as the rate of change in depth over a one second period, was used to split the depth record into vertical swimming phases (“ascending”, “descending” and “level swimming”). This was executed by smoothing the depth record using a 10 s running mean and calculating the average VV by taking the difference of this smoothed depth between successive points at 1 s intervals. Ascents and descents were defined where VV exceeded an absolute value of 0.05 m/s for more than 10 s, and level where this value was not exceeded [1, 2]. As error in the depth sensor was minimal (±10 cm), we do not believe sensor accuracy significantly affected vertical movement phase classification.

Recovery period:

Tailbeat data were used to calculate the recovery period from capture using metrics of tailbeat activity following methods outlined by Whitney et al. [2]. Briefly, tailbeat cycle (the inverse of tailbeat frequency) throughout descent was summarized for 15 minute windows, and plotted against time post-release. A recovery period was defined as the time it took for this metric to reach 80% of its asymptote [2]. This was calculated for all tiger sharks, with the exception of two individuals that had tag deployment durations of <5 h. The overall mean recovery period was then eliminated from the tri-axial sensor data prior to further analyses to remove potential sublethal and unnatural behaviors resulting from stress of capture by drumlines.

Gliding behaviour

We used a continuous wavelet transformation on the dynamic component of the sway (i.e. lateral) axis to calculate the signal amplitude and frequency of shark tailbeats using the angular velocity data [3, 7]. These data were used to quantify the incidence of gliding behaviour – defined here as a cessation of tailbeats for more than one second – through a two-step process as per Andrzejaczek et al. [1]. Briefly, (1) gliding behaviour was isolated for each individual shark using the 'k-means cluster' function in the Ethographer for Igor Pro [3]. This function clustered the spectra computed by the wavelet transformation based on similarity of shape. The behavioural spectrum with the lowest peaks in angular velocity signal amplitude was assumed to represent gliding behaviour [8], and the incidence of the resulting cluster was then inspected against the dynamic sway data. As this cluster did not match with gliding behaviour in some individuals (i.e. tailbeats evident in sway data were classified to be gliding, and vice versa), (2) threshold values of angular velocity signal amplitude and tailbeat frequency were set using the characteristics of correctly classified gliding behavior (from visual inspection of the dynamic sway data and concurrent videos). These thresholds were then used to extract glides from all sharks, and an additional manual quality control was undergone in the case where the mask obviously misclassified glides (Figure S1).

Ascent and descent speeds

Vertical velocity (VV) and pitch (φ) were used to estimate the mean speed of ascents and descents through trigonometry as per:

$$Speed\ (ms^{-1}) = \frac{Vertical\ velocity\ (ms^{-1})}{\sin(\varphi)}$$

This, however, could only be calculated when pitch exceeded 20° due to the large errors associated with estimating speed at low pitch angles [9].

Window size and statistics

The sampling window used for analysis was determined by calculating the time period where the highest variance in turning angles was observed, while being of sufficient size to capture the longest recorded dives in their entirety at all depths as per Andrzejaczek et al. [7]. This time window was estimated to be 15 minutes here (900 seconds; Figure S1, S2), though we estimate in habitats deeper than encountered here, this would need to be extended. Therefore, a number of vertical movement parameters were summarized for each 15 minute window of each deployment including mean (± standard deviation) and maximum depth, ascent pitch, descent pitch, ascent VV and descent VV. The percent of time spent moving

vertically (ascending and descending), termed the 'diving ratio', was also calculated within each window for each individual as per:

$$Diving\ ratio = \frac{Time\ vertically\ moving\ in\ window\ (seconds)}{Total\ time\ in\ sampling\ window\ (900\ seconds)}$$

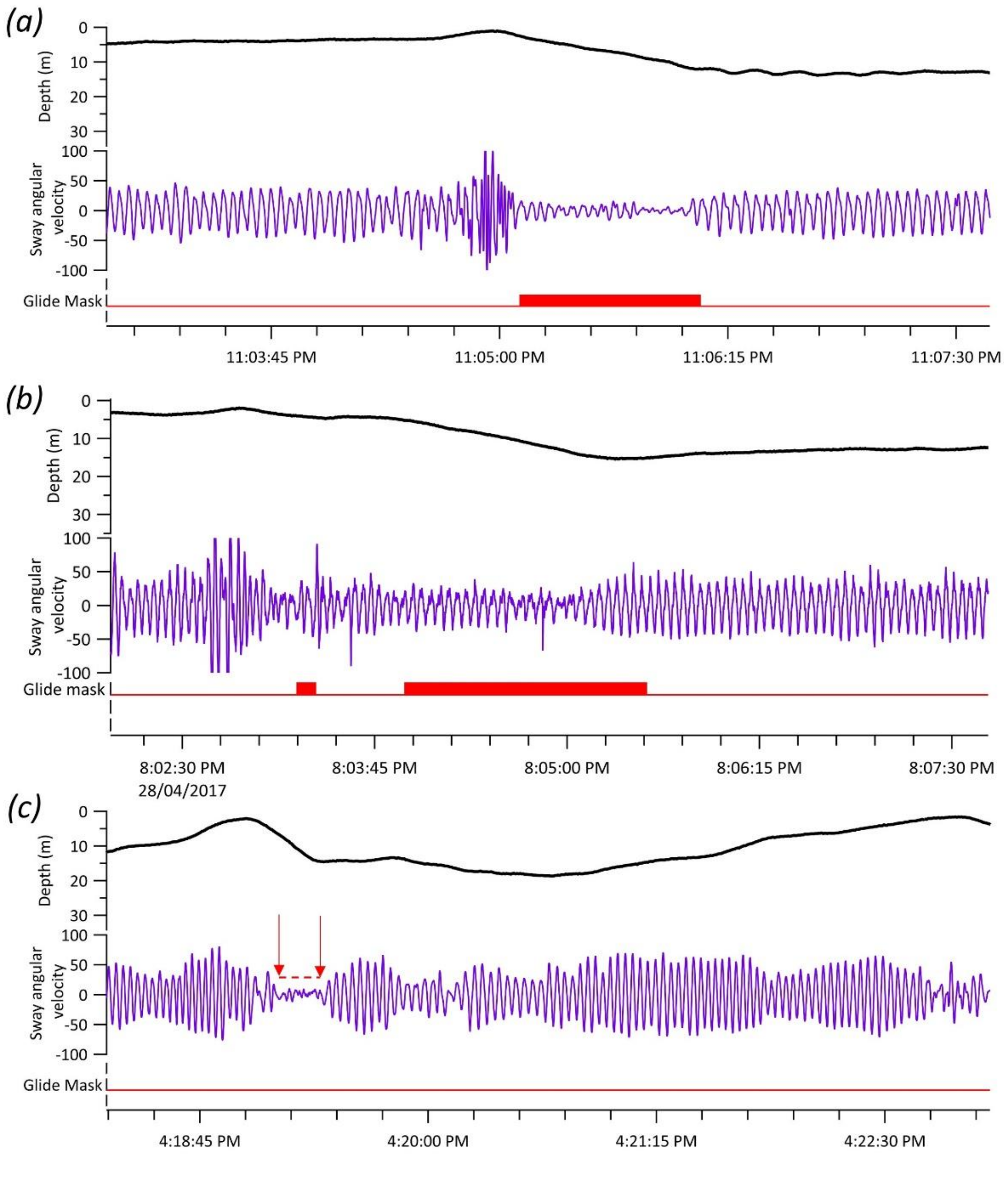

Figure S1. Examples of mask classification encountered throughout manual quality control: (a) a correctly classified glide; (b) an active descent wrongly classified as a glide; (c) a glide not classified as such by the mask.

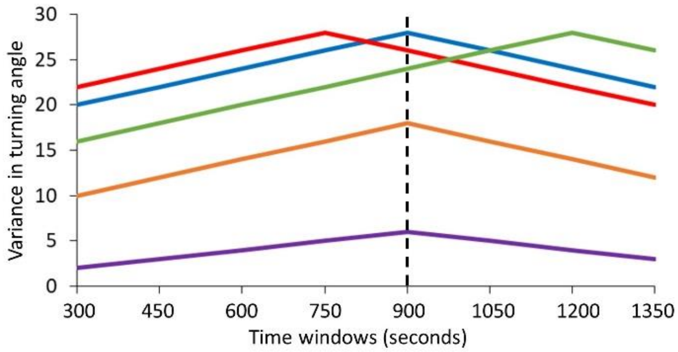

Figure S2. Schematic diagram of how variance in turning angle was investigated among individual tiger sharks. Each coloured line represents an individual shark. Dashed line at 900 seconds (15 minutes) demonstrates overlap with highest number of individuals.

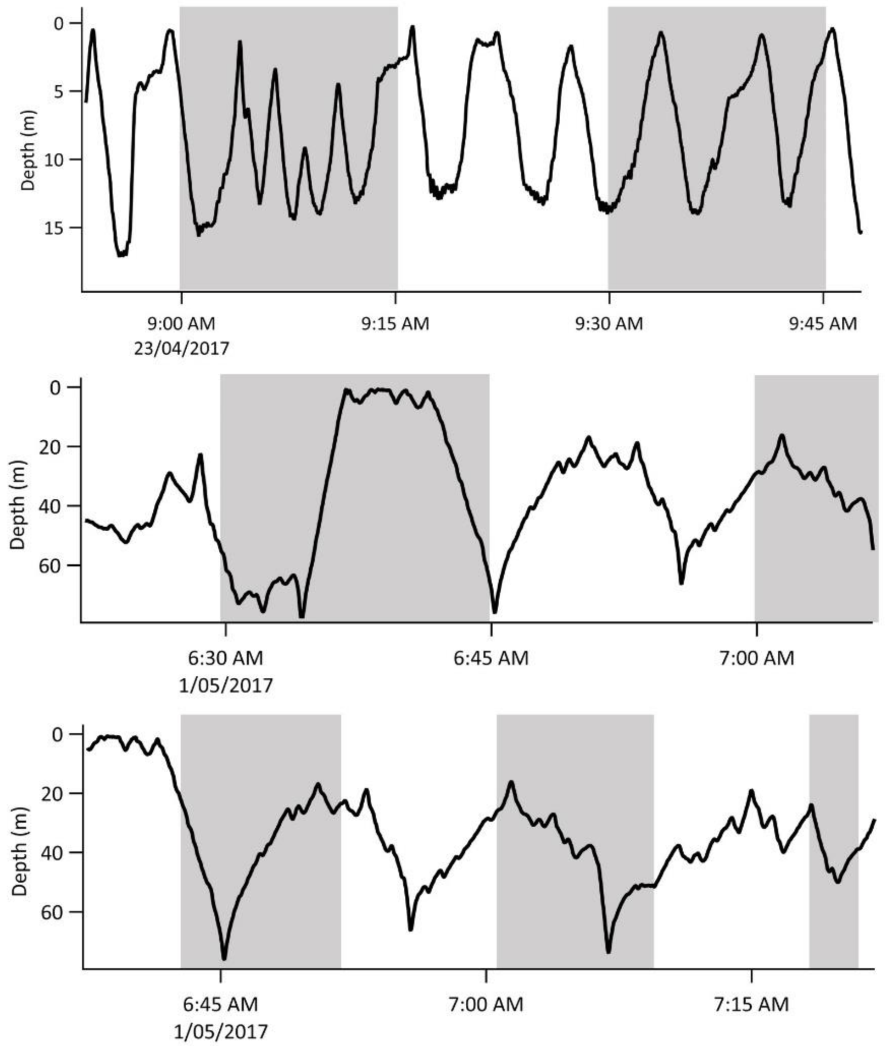

Figure S3. Depth time-series for three different individuals moving in three different depth zones. Grey and white bars indicate 15-minute sampling windows, and demonstrate dives recorded in entirety in both shallow and deep habitats.

Data analysis:

Generalised linear mixed models (GLMMs)

Generalised linear mixed models (GLMMs) were built using the `nlme` package in R 3.4.0 [10, 11] to investigate possible relationships between seabed depth and vertical movement behaviours in tiger sharks. The maximum depth (m) recorded within each time window was used as a proxy for seabed depth (based on video analysis; see Andrzejaczek *et al.* [12]) and was set as the explanatory variable, and tiger shark identity set as a random variable for all models. Ascent pitch, descent pitch, ascent VV, descent VV and diving ratio were all set sequentially as response variables. We used the corAR1 function to account for temporal auto-correlation in our datasets [13]. Together with nautical charts from Ningaloo Reef, maximum depth was used to classify windows as either 'inshore' (<25 m, inside the reef) or 'offshore' (>25 m, outside the reef). GLMMs were analysed separately for inshore and offshore periods due to heterogeneity in residuals and an unbalanced design. The resulting models were compared against the null models and ranked using Akaike's information criterion (AIC).

To investigate if observed changes in diving ratio with depth were an artifact of our selected sampling window, we calculated diving ratio for oscillations occurring in increasingly deeper water given a fixed interval of level swimming at the surface and seabed. One-hour long depth traces were simulated for a hypothetical shark oscillating in depths of 5, 10, 20, 30, 40, 50 and 60 m. The ascent VV and descent VV for each depth zone were determined following relationships calculated between VV and seabed depth (see above). The fixed interval spent at the surface and on the seabed between vertical movements was set at two minutes following exploration of the depth traces. Diving ratio was calculated for each of the four 15 minute windows, and averaged for the hour so that one value of diving ratio was calculated for each depth.

Cost of transport models

We modelled the cost of transport of oscillatory movements of varying geometries in relation to optimization of either horizontal or vertical distance travelled following methods similar to those described by Gleiss *et al.* [9] (figure 1). First, we calculated the total mechanical cost (TC) of an oscillation (an ascent (a) and descent (d) combined) in units of ODBA (g) using the equation:

$$\mathrm{TC} = \mathrm{T_a} \times \mathrm{ODBA_a} + \mathrm{T_d} \times \mathrm{ODBA_d} + \mathrm{k} \times (\mathrm{T_d} + \mathrm{T_a})$$

Where T_a and T_d are the time spent ascending and descending, respectively, $ODBA_a$ and $ODBA_d$ are the ODBA of ascents and descents, respectively, and k is a proxy for basal metabolic cost. Previous studies have shown that basal metabolic costs are approximately 60% of routine metabolic rate in sharks [see 14 and references therein], and therefore we estimated k at 60% of the mean ODBA recorded for all sharks (0.026 g) or k = 0.0156 g. T_a, T_d, $ODBA_a$, and $ODBA_d$, were all calculated depending on pitch angle (φ). $ODBA_a$ was estimated from the quadratic relationship between ODBA and φ_a (figure 2A; ODBA = 2E-05 φ_a^2 + 0.00001φ_a + 0.0226), and for $ODBA_d$, a single mean value of ODBA during descents (0.012 g) was used, as no relationship was found between φ_d and ODBA (figure 2B). T_a and T_d are a function of φ_a and φ_d, respectively, depth, and mean speed, and were calculated using the following equations:

$$T_a = \frac{\frac{Depth}{\sin(\varphi_a)}}{\text{mean ascent speed}}$$

$$T_d = \frac{\frac{Depth}{\sin(\varphi_d)}}{\text{mean descent speed}}$$

We used a fixed mean estimate of speed for both ascents (0.87 m s^{-1}) and descents (0.85 m s^{-1}) as no relationship was found between speed and pitch angle (Table S2). TC was calculated for fixed ascent angles from 5° to 45°, binned in 5° increments. For each bin of ascent angles, TC was calculated sequentially for decent angles of 5° to 20°, binned in 5° increments.

We constructed two different models describing the cost of horizontal transport (COT_{HD}) and cost of vertical transport (COT_{VD}) for tiger sharks. These models calculated the cost of moving a unit of horizontal (HD) and vertical distance (VD) respectively, and were used to determine the angles that optimized the efficiency of transport on each of these scales. The COT_{HD} was modelled by:

$$COT_{HD} = \frac{TC}{HD}$$

Where horizontal distance was calculated from ascent and descent pitch using the equation:

$$HD = \frac{Depth}{\tan(\varphi_d)} + \frac{Depth}{\tan(\varphi_a)}$$

The COT_{VD} was modelled by:

$$COT_{VD} = \frac{TC}{2 \times Depth}$$

All model calculations used oscillations of 10 m depth, however, the resulting COT for horizontal and vertical distance was the same regardless of depth.

Table S2. Estimated ascent and descent speeds calculated by pitch and vertical velocity for absolute dive angles >20° for each individual tiger shark, and their relationship with pitch. Individuals with <20 points were excluded (TS10, TS16, TS20, TS25). Note that where significant relationships exist, slopes do not exceed an absolute value of 0.02.

Shark ID	Vertical phase	N	Mean speed	F-value	P	r^2	Slope	Intercept
TS1	Ascent	58	0.78	3.7	0.06	0.06	-0.01	1.04
	Descent	540	0.79	0.41	0.52	0.008	<-0.001	-0.8
TS2	Ascent	181	0.87	1.70	0.19	0.009	-0.003	0.96
	Descent	281	0.86	59.2	<0.001	0.17	-0.01	0.56
TS3	Ascent	24	0.79	17.1	<0.001	0.43	-0.008	0.99
	Descent	88	0.87	3.6	0.06	0.04	-0.006	0.71
TS4	Ascent	215	0.91	8.8	0.003	0.04	0.01	0.59
	Descent	315	0.90	10.36	0.001	0.03	-0.018	0.45
TS5	Ascent	504	0.97	5.8	0.02	0.01	0.004	0.87
	Descent	979	0.82	21.59	<0.001	0.02	-0.003	0.75
TS6	Ascent	85	0.72	34.6	<0.001	0.3	0.009	0.49
	Descent	456	0.58	41.02	<0.001	0.08	-0.007	0.41
TS8	Ascent	294	0.89	6.24	0.01	0.02	0.004	0.77
	Descent	716	0.69	72.3	0.001	0.09	-0.01	0.40
TS9	Ascent	858	1.03	38.87	<0.001	0.04	0.004	0.92
	Descent	1580	1.0	3157	0.001	0.67	-0.02	0.43
TS12	Ascent	50	0.65	2.1	0.155	0.04	-0.002	0.71
	Descent	112	0.70	0.001	0.97	<0.001	-0.0001	0.69
TS13	Ascent	2041	0.92	3.93	0.05	0.002	-0.0007	0.94
	Descent	3439	0.96	2455	0.001	0.42	0.02	0.4
TS14	Ascent	216	0.91	0.06	0.81	0.0003	-0.0007	0.92
	Descent	301	0.83	0.17	0.68	0.0005	-0.002	0.79
TS15	Ascent	1149	0.74	9.5	0.002	0.008	0.002	0.67
	Descent	1362	0.76	181.6	<0.001	0.12	-0.009	0.53
TS17	Ascent	127	0.98	0.52	0.47	0.004	0.002	0.94
	Descent	663	0.96	46.2	<0.001	0.07	-0.02	0.56
TS18	Ascent	1059	1.25	11.48	<0.001	0.01	-0.003	1.3
	Descent	3115	1.1	373.6	<0.001	0.11	-0.01	0.84
TS19	Ascent	101	0.90	13.02	<0.001	0.12	0.01	0.62
	Descent	78	0.73	5.57	0.02	0.07	-0.005	0.60
TS24	Ascent	63	0.98	1.8	0.18	0.03	0.01	0.67

	Descent	263	0.89	15.4	<0.001	0.06	-0.005	0.75
TS27	Ascent	958	0.76	26.3	<0.001	0.03	0.004	0.66
	Descent	118	1.03	49.4	<0.001	0.31	-0.02	0.21

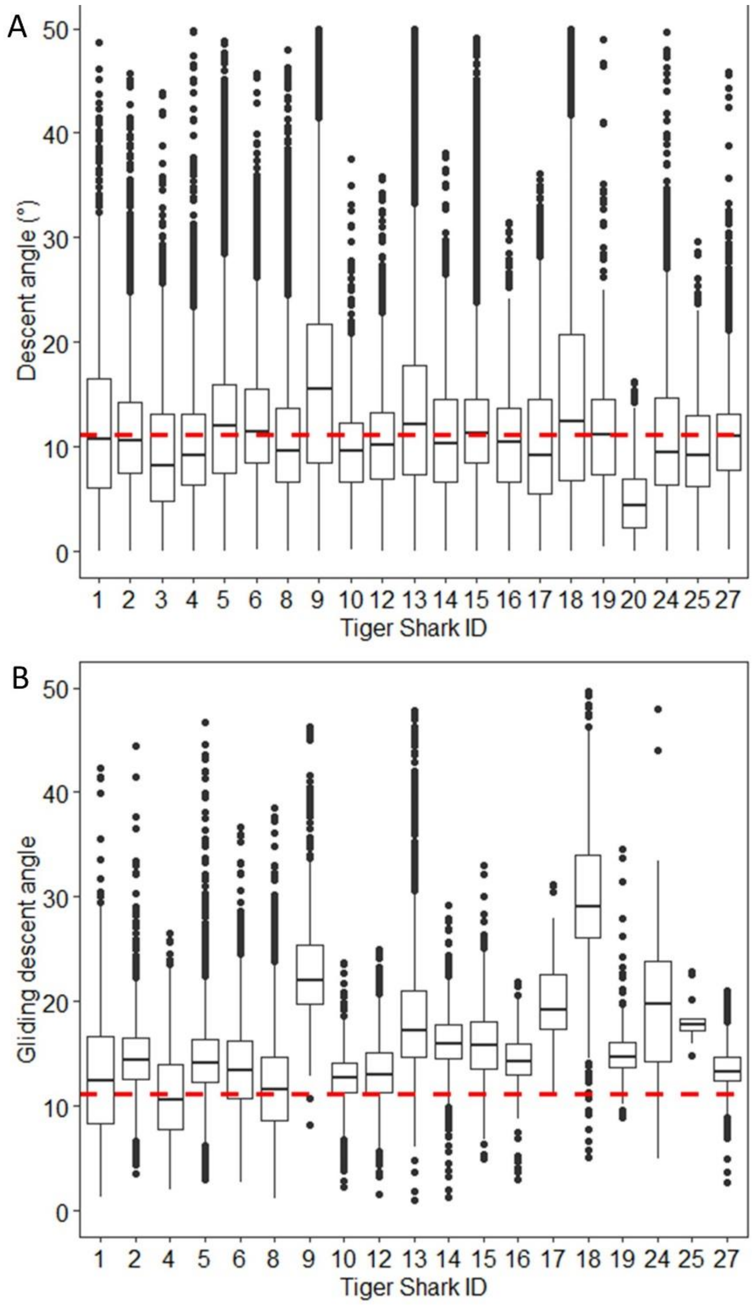

Figure S4. Individual differences in absolute angles on descent for A) all descent data, and B) passive gliding descents only. Note that in B), TS3 and TS20 are not present due to no gliding behaviour being exhibited by these two individuals. The red dashed line indicates the mean descent angle (11.1°). Also note that TS17 and TS24 are the same individual, tagged 11 days apart).

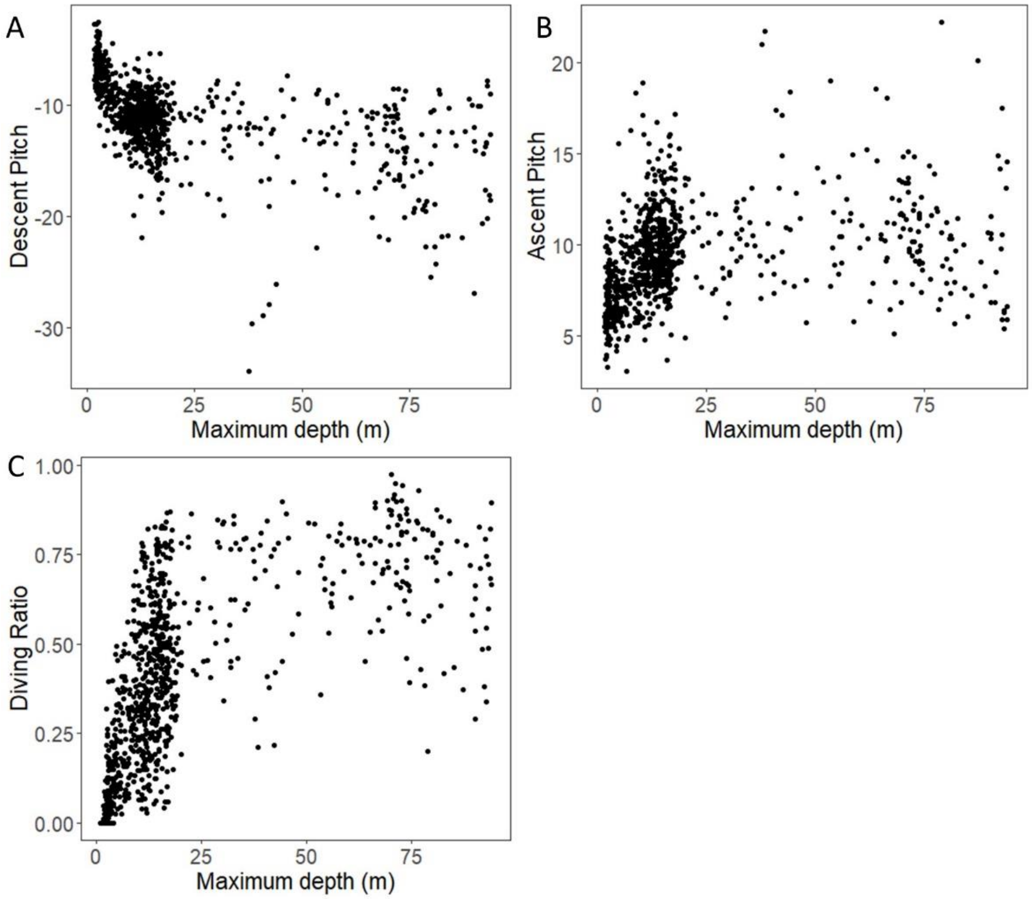

Figure S5. Relationships between vertical movement behaviours and maximum depth; (A) Maximum depth and ascent angle for all data. (B) Maximum depth and descent angle for all data. (C) Maximum depth and diving ratio for all data.

Reference list:

1 Andrzejaczek, S., Gleiss, A. C., Pattiaratchi, C. B., Meekan, M. G. 2018 First insights into the fine-scale movements of the sandbar shark, *Carcharhinus plumbeus*. *Frontiers in Marine Science*. **5**, (10.3389/fmars.2018.00483)

2 Whitney, N. M., White, C. F., Gleiss, A. C., Schwieterman, G. D., Anderson, P., Hueter, R. E., Skomal, G. B. 2016 A novel method for determining post-release mortality, behavior, and recovery period using acceleration data loggers. *Fisheries Research*. **183**, 210-221. (http://dx.doi.org/10.1016/j.fishres.2016.06.003)

3 Sakamoto, K. Q., Sato, K., Ishizuka, M., Watanuki, Y., Takahashi, A., Daunt, F., Wanless, S. 2009 Can ethograms be automatically generated using body acceleration data from free-ranging birds. *PLoS one*. **4**, e5379. (10.1371/journal.pone.0005379)

4 Shepard, E. L. C., Wilson, R. P., Halsey, L. G., Quintana, F., Laich, A. G., Gleiss, A. C., Liebsch, N., Myers, A. E., Norman, B. 2008 Derivation of body motion via appropriate smoothing of acceleration data. *Aquatic Biology*. **4**, 235-241. (10.3354/ab00104)

5 Kawatsu, S., Sato, K., Watanabe, Y., Hyodo, S., Breves, J. P., Fox, B. K., Grau, E. G., Miyazaki, N. 2009 A new method to calibrate attachment angles of data loggers in swimming sharks. *EURASIP Journal on Advances in Signal Processing*. **2010**, 732586. (10.1155/2010/732586)
6 Wilson, R. P., White, C. R., Quintana, F., Halsey, L. G., Liebsch, N., Martin, G. R., Butler, P. J. 2006 Moving towards acceleration for estimates of activity-specific metabolic rate in free-living animals: the case of the cormorant. *Journal of Animal Ecology*. **75**, 1081-1090. (10.1111/j.1365-2656.2006.01127.x)
7 Andrzejaczek, S., Gleiss Adrian, C., Lear, K. O., Pattiaratchi, C. B., Chapple Taylor, K., Meekan, M. G. 2019 Biologging Tags Reveal Links Between Fine-Scale Horizontal and Vertical Movement Behaviors in Tiger Sharks (*Galeocerdo cuvier*). *Frontiers in Marine Science*. **6**, 1:13. (10.3389/fmars.2019.00229)
8 Nakamura, I., Watanabe, Y. Y., Papastamatiou, Y. P., Sato, K., Meyer, C. G. 2011 Yo-yo vertical movements suggest a foraging strategy for tiger sharks *Galeocerdo cuvier*. *Marine Ecology Progress Series*. **424**, 237-246. (10.3354/meps08980)
9 Gleiss, A. C., Norman, B., Wilson, R. P. 2011 Moved by that sinking feeling: variable diving geometry underlies movement strategies in whale sharks. *Functional Ecology*. **25**, 595-607. (10.1111/j.1365-2435.2010.01801.x)
10 Pinheiro, J., Bates, D., DebRoy, S., Sarkar, D., Heisterkamp, S., Van Willigen, B., Maintainer, R. 2017 Package 'nlme'. *Linear and Nonlinear Mixed Effects Models, version*. 3-1.
11 R Core Team. R: A Language and Environment for Statistical Computing. In: R. F. f. S. Computing, ed. Vienna, Austria 2017.
12 Andrzejaczek, S., Gleiss, A. C., Lear, K. O., Pattiaratchi, C. B., Chapple, T. K., Meekan, M. G. 2019 Biologging Tags Reveal Links Between Fine-Scale Horizontal and Vertical Movement Behaviors in Tiger Sharks (*Galeocerdo cuvier*). *Frontiers in Marine Science*. **6**, (10.3389/fmars.2019.00229)
13 Zuur, A., Ieno, E. N., Walker, N., Saveliev, A. A., Smith, G. M. 2009 *Mixed effects models and extensions in ecology with R*. Springer Science & Business Media.
14 Dowd, W. W., Brill, R. W., Bushnell, P. G., Musick, J. A. 2006 Standard and routine metabolic rates of juvenile sandbar sharks (*Carcharhinus plumbeus*), including the effects of body mass and acute temperature change. *Fishery Bulletin*. **104**, (http://escholarship.org/uc/item/9g59202m)
